# Fast phonetic learning occurs already in 2-to-3-month old infants: an ERP study

**DOI:** 10.3389/fpsyg.2014.00077

**Published:** 2014-02-25

**Authors:** Karin Wanrooij, Paul Boersma, Titia L. van Zuijen

**Affiliations:** ^1^Amsterdam Center for Language and Communication, University of AmsterdamAmsterdam, Netherlands; ^2^Department of Child Development and Education, University of AmsterdamAmsterdam, Netherlands

**Keywords:** distributional learning, infant MMR (mismatch response), perceptual asymmetry, language acquisition, category learning, ERP, speech perception

## Abstract

An important mechanism for learning speech sounds in the first year of life is “distributional learning,” i.e., learning by simply listening to the frequency distributions of the speech sounds in the environment. In the lab, fast distributional learning has been reported for infants in the second half of the first year; the present study examined whether it can also be demonstrated at a much younger age, long before the onset of language-specific speech perception (which roughly emerges between 6 and 12 months). To investigate this, Dutch infants aged 2 to 3 months were presented with either a unimodal or a bimodal vowel distribution based on the English /æ/~/ε/ contrast, for only 12 minutes. Subsequently, mismatch responses (MMRs) were measured in an oddball paradigm, where one half of the infants in each group heard a representative [æ] as the standard and a representative [ε] as the deviant, and the other half heard the same reversed. The results (from the combined MMRs during wakefulness and active sleep) disclosed a larger MMR, implying better discrimination of [æ] and [ε], for bimodally than unimodally trained infants, thus extending an effect of distributional training found in previous behavioral research to a much younger age when speech perception is still universal rather than language-specific, and to a new method (using event-related potentials). Moreover, the analysis revealed a robust interaction between the distribution (unimodal vs. bimodal) and the identity of the standard stimulus ([æ] vs. [ε]), which provides evidence for an interplay between a perceptual asymmetry and distributional learning. The outcomes show that distributional learning can affect vowel perception already in the first months of life.

## INTRODUCTION

Distributional learning, i.e., learning by simply being exposed to the frequency distributions of stimuli in the environment, may be one of the mechanisms by which infants start to acquire the phonemes of their language (Lacerda, 1995; Guenther and Gjaja, 1996). *Fast* distributional learning of speech sounds after just a few minutes of exposure in the lab has been observed in infants in the second half of the first year (e.g., Maye et al., 2008). This study investigates whether such fast distributional learning can also take place in very young infants, i.e., 2-to-3-month olds. This is relevant if we want to establish that the distributional learning mechanism is in place early enough to be able to contribute to the transition from universal to language-specific speech perception, which becomes apparent in infants’ speech sound discrimination from around 6 months of age (e.g., Werker and Tees, 1984/2002; Polka and Werker, 1994), or perhaps even from 4 months (Yeung et al., 2013).

In the first year of life, infants’ speech sound perception has been observed to change from universal to language-specific. Specifically, in the course of this transition discrimination performance is enhanced for native speech sound contrasts (Cheour et al., 1998b; Kuhl et al., 2006; Tsao et al., 2006), and reduced for non-native contrasts that are irrelevant in the native language (Werker and Tees, 1984/2002; Kuhl et al., 1992; Tsushima et al., 1994; Polka and Werker, 1994; Best et al., 1995; Bosch and Sebastián-Gallés, 2003; Kuhl et al., 2006; Tsao et al., 2006). In general, language-specific speech sound discrimination emerges between 4 and 6 months for tones (i.e., in tonal languages; Cheng et al., 2013; Yeung et al., 2013), around 6 months for vowels (Kuhl et al., 1992; Polka and Werker, 1994; Bosch and Sebastián-Gallés, 2003), and between 8 and 12 months for consonants (Werker and Tees, 1984/2002; Tsushima et al., 1994; Best et al., 1995; Kuhl et al., 2006; Tsao et al., 2006), although language-specific discrimination of difficult contrasts may develop later (e.g., Cheour et al., 1998b; Polka et al., 2001; Sundara et al., 2006).

One of the mechanisms that has been hypothesized to contribute to the emergence of language-specific speech perception is distributional learning (Lacerda, 1995; Guenther and Gjaja, 1996). The existence of this mechanism has indeed been supported by observations in the lab. In particular, fast distributional learning has been demonstrated most reliably in 8-month olds by Maye et al. (2008; *p *< 0.001), and (nearly) significantly in 6-to-8-month olds by Maye et al. (2002; *p* = 0.063), in 10-to-11-month olds by Yoshida et al. (2010; *p* = 0.036 for one of the experiments), and in 11-month olds by Capel et al. (2011; *p* = 0.053), although null results were found in 10-to-11-month olds by Yoshida et al. (2010; for two experiments) and ambiguous results were found in 5-month olds by Cristià et al. (2011; *p *> 0.16 for the main effect, but *p* = 0.007 for an interaction effect).

If distributional learning indeed contributes to the acquisition of language-specific perception, and discriminational evidence for the latter starts being observed from 4 or 6 months on, fast distributional learning can be expected to be detectable in even younger infants. This expectation is supported by neuroscientific research. Cortical layers involved in top-down processing (e.g., Kral and Eggermont, 2007) become anatomically available in humans from around 4 to 5 months of age (Moore and Guan, 2001; Moore, 2002; Moore and Linthicum, 2007), which suggests that speech perception before 4 months relies mainly on bottom-up processing. The distributional learning mechanism, which supposedly does not require top-down processing (Guenther and Gjaja, 1996), should therefore at this early age be relatively unimpeded by learning mechanisms that require top-down influence from higher-level (e.g., lexical) representations.

**FIGURE 1:**
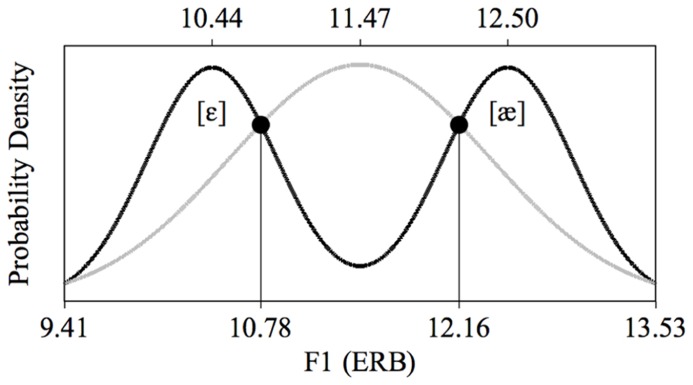
**Unimodal (gray curve) and bimodal (black curve) training distributions of the first vowel formant (F1).** The values of the test stimuli lie at the intersections of the two distributions.

We therefore performed a fast distributional learning experiment with infants aged 2 to 3 months. Specifically, we presented Dutch infants of this age with speech sounds from an acoustic continuum encompassing the British-English vowel contrast /æ/~/ε/; this is a contrast that does not exist in Dutch, and which Dutch adults find difficult to master (e.g., Schouten, 1975; Weber and Cutler, 2004; Broersma, 2005; Escudero et al., 2008). These vowels differ in their first formant (F1), as illustrated in **Figure 1**, where the F1 values are given in ERB (Equivalent Rectangular Bandwidth; see section Stimuli for details). In our experiment, one half of the infants were exposed to a *unimodal distribution* (**Figure 1**, gray), i.e., to a large number of different vowel tokens whose F1 values center around 11.47 ERB, which is phonetically halfway between English [ε] and [æ], and the other half of the infants were exposed to a *bimodal distribution* (**Figure 1**, black), i.e., to a large number of vowel tokens whose F1 values center around 10.44 and 12.50 ERB, which are F1 values typical of English [ε] and [æ], respectively. The bimodal distribution thus suggests the existence of a contrast between /æ/ and /ε/ (as would be appropriate for learners of English), while the unimodal distribution does not suggest a contrast between the two vowels (as would be appropriate for learners of Dutch). Immediately after the training we tested how well the infants discriminated an open variant of English [ε], i.e., a vowel with an F1 of 10.78 ERB, and a closed variant of English [æ], i.e., a vowel with an F1 of 12.16 ERB, both visible in **Figure 1**. If distributional learning occurred, bimodally trained infants should discriminate them better than unimodally trained infants.

Discrimination ability after training had to be measured with a method appropriate for young infants. All previous research on infant or adult distributional learning employed behavioral measures, which for infants always meant looking time. Since suitable behavioral responses are difficult to obtain from 2-to-3-month olds, we instead measured an automatic brain response, namely the *mismatch response *(MMR; e.g., Näätänen et al., 1978). In contrast to behavioral measurements, which require the infant’s cooperation and attention (Cheour et al., 2000, p. 6), the MMR is elicited even in the absence of voluntary attention to the stimuli (e.g., Schröger, 1997; Näätänen and Winkler, 1999), and can be measured even when the infant is asleep (Friederici et al., 2002; Martynova et al., 2003). The MMR has been shown to reflect behavioral discrimination in adults (for a review, see Näätänen et al., 2007), and has been used successfully before to demonstrate vowel discrimination in infants of 3 months and younger (e.g., Cheour-Luhtanen et al., 1995; Cheour et al., 1998a; Martynova et al., 2003; Kujala et al., 2004; Shafer et al., 2011; Partanen et al., 2013). The MMR can be elicited in an oddball paradigm (e.g., Näätänen, 1992), where a series of “standard” stimuli (e.g., [ε] tokens) is interspersed infrequently with “deviant” stimuli (e.g., [æ] tokens). If the auditory perception system detects that deviants differ from standards, it will process the two kinds of stimuli in different ways, which can be reflected in the event-related potentials (ERPs). The MMR can be computed as the difference between the ERP elicited by the deviants and the ERP elicited by the standards.

When measuring MMRs to speech sounds in an oddball paradigm, it can make a difference whether one or the other stimulus of a pair is chosen as a standard. Possible asymmetries in participants’ perception can exist, which can make discrimination easier if one particular stimulus (e.g., [æ]) is the standard than if the contrasting stimulus (e.g., [ε]) is the standard. Perceptual biases have been reported for several speech sounds (Pisoni, 1977; Aslin and Pisoni, 1980; Polka and Bohn, 1996, 2003) and seem especially strong in young infants (Pons et al., 2012). For vowels one relevant perceptual bias is a peripherality-related asymmetry: when hearing a more peripheral vowel after a more central vowel (i.e., in a two-dimensional acoustic space defined by the first and second vowel formants) discrimination is easier than when hearing the same vowels in the opposite order (e.g., Polka and Bohn, 1996, 2003; Pons et al., 2012). This would predict that in our oddball paradigm discrimination may be easier if [ε] is the standard stimulus than if [æ] is the standard. Further, the “natural referent vowel” hypothesis (Polka and Bohn, 2011) predicts that this perceptual bias will vanish or grow fainter for native contrasts and will remain or grow stronger for non-native contrasts. This would predict that if fast distributional training already leads to some sort of vowel category formation, unimodally trained infants, for whom the contrast /æ/~/ε/ is new (“non-native”), will show a perceptual asymmetry, whereas the bias will not be clear in bimodally trained infants, for whom the contrast is experienced during training (“native”). Other perceptual biases can be expected on the basis of hypotheses involving underspecification (Lahiri and Reetz, 2010), according to which a featurally underspecified phoneme will mismatch with a preceding specified phoneme, but the reverse order will not lead to a mismatch. This would predict that if [æ] is specified for the feature [low] and [ε] is not, discrimination may be easier if [æ] is the standard stimulus than if [ε] is the standard. To accommodate the main and interaction effects of any perceptual biases, we counterbalanced the identity of the standard ([æ] or [ε]) across the infants and included it as a factor in the analysis.

In sum, the aim of the current study was to investigate whether 2-to-3-month old infants already show fast distributional learning, by training Dutch infants of this age on either a unimodal or a bimodal distribution of the English vowel contrast /æ/~/ε/, and then testing in an ERP oddball paradigm how well they discriminate [æ] from [ε]. If the distributional learning mechanism exists, it is expected that bimodally trained infants, who hear a distribution that suggests the existence of a contrast between /æ/ and /ε/, discriminate [æ] and [ε] better, and thus have a *larger MMR amplitude*, than unimodally trained infants, who hear a distribution that does not suggest a contrast between /æ/ and /ε/.

## MATERIALS AND METHODS

### PARTICIPANTS

The 32 infants (11 girls) accepted for the study met the following criteria. The language spoken at home had to be Dutch only. The infant had to be healthy and had to have passed the Dutch otoacoustic emissions test for newborns. Birth weight had to be normal (each infant weighed over 2500 g). The Apgar score had to be 8 or higher 10 minutes after birth. The gestational age at birth had to be between 37 and 42 weeks, and the post-natal age from birth to time of testing between 8 and 12 weeks. Finally, we excluded infants born with complications, but accepted infants delivered by Caesarean section. The study protocol was approved by the Ethical Committee of the Faculty of Social and Behavioral Sciences at the University of Amsterdam. Parents signed informed consent forms.

### DESIGN

All infants listened to a training distribution and performed a subsequent discrimination test. During the training, half of the infants heard a bimodal distribution, with peaks around [æ] and around [ε], and the other half a unimodal distribution, with a single broad peak between [æ] and [ε]. During the test, half of the infants in each distributional training group listened to standard [æ] and deviant [ε], and the other half to standard [ε] and deviant [æ]. Thus, based on Distribution Type (unimodal vs. bimodal) and Standard Vowel ([æ] vs. [ε]) the 32 infants were assigned to four “groups,” namely Unimodal [æ], Unimodal [ε], Bimodal [æ], and Bimodal [ε], each consisting of eight infants. Apart from balancing the sexes, assignment to the groups was random.

After separating the data into non-quiet sleep (non-QS) and quiet sleep (QS) data (section Coding Sleep Stages) and applying a criterion for a sufficient number of valid responses (section ERP Recording and Analysis), we could include the non-QS data of 22 infants in the non-QS dataset, and the QS data of 21 infants in the QS dataset (12 infants contributed to both datasets, 19 to one dataset, and one to no dataset). In the non-QS dataset the number of contributing infants was five in Unimodal [æ], six in Unimodal [ε], six in Bimodal [æ], and five in Bimodal [ε]. In the QS dataset the number of contributing infants was six in Unimodal [æ], four in Unimodal [ε], five in Bimodal [æ], and six in Bimodal [ε].

To sum up, the experimental design for measuring the effect of distributional training had Distribution Type (unimodal vs. bimodal) and Standard Vowel ([æ] vs. [ε]) as between-subject factors, and the MMR amplitude as the dependent variable, to be determined separately for the QS and the non-QS dataset.

### STIMULI

Test and training stimuli were made with the Klatt synthesizer in the computer program Praat (Boersma and Weenink, 2010) and varied only in the values for the first and second formants, F1 and F2 (see sections In the Training and In the Test). The duration of each stimulus was kept at 100 ms (e.g., Cheour-Luhtanen et al., 1995; Cheour et al., 1998a, 2002b) including rise and fall times of 5 ms. The fundamental frequency contour fell from 150 to 112.5 Hz, which represents a male voice (e.g., Cheour-Luhtanen et al., 1995; Cheour et al., 1998a, 2002b; Martynova et al., 2003). The source signal was filtered with eight additional formants (F3 through F10). The values for F3, F4, and F5, which were 2400, 3400, and 4050 Hz respectively, were extracted from American-English vowels representing /æ/ and /ε/ in the TIMIT database (Lamel et al., 1986), while those for F6 through F10 were calculated as the previous formant plus 1000 Hz (e.g., F6 = F5 + 1000 Hz). Similarly, the bandwidth values for the first four bandwidths, which were 80, 160, 360, and 530 Hz, respectively, were based on the TIMIT database, while an additional six bandwidths were calculated as the corresponding formant divided by 8.5 (e.g., bandwidth 5 = F5/8.5). Each stimulus was made equally loud, to avoid possible confounds in the ERPs based on intensity differences (Näätänen et al., 1989; Sokolov et al., 2002). The stimuli were played (during training and test) at around 70 dB SPL, measured at about one meter from the two loudspeakers, where the infant was lying.

#### In the training

The unimodal and bimodal training distributions were created in the manner reported by Wanrooij and Boersma (2013). In contrast with previous research, which typically employed only eight different stimulus values, each of which was repeated multiple times during training, this method uses more ecologically valid *continuous* training distributions, where all presented stimuli are acoustically different. Each of the two distributions thus consisted of 900 unique vowels and had an identical range of F1 and F2 values: 9.41 to 13.53 ERB for F1 and 21.05 to 18.31 ERB for F2 (see also **Figure 1**). These ranges were based on values for F1 and F2 as reported by Hawkins and Midgley (2005). Specifically, we took the reported F1 and F2 values of /æ/ and /ε/, each pronounced four times by five male speakers of British English in the age group 35–40 years, and converted the hertz values to ERB. Hawkins and Midgley’s mean F1 and F2 were 12.51 ERB and 18.94 ERB, respectively for /æ/, and 10.43 ERB and 20.42 ERB for /ε/. Because in the current study the stimuli were produced by one synthetic speaker, a single-speaker standard deviation, for F1 and F2 separately, was calculated as the mean of the five speakers’ standard deviations for the vowel /ε/. The standard deviations were 0.51 ERB for F1 and 0.32 ERB for F2. The edges of the F1 and F2 ranges, mentioned above, were determined to lie two standard deviations from the mean F1 and F2 values of the vowels; for instance, the lower edge of the F1 continuum lay at 10.43 - 2 × 0.51 = 9.41 ERB. Note that in going from /ε/ to /æ/ the F1 rises, while F2 declines.

The shape of the distributions was defined in accordance with earlier distributional learning studies in that the ratio of the least to most frequent stimuli was about 1 to 4 (e.g., Maye et al., 2002, 2008). As illustrated in **Figure 1**, the unimodal mean lay exactly in the middle of the range of F1 (or F2) values and precisely in between the two bimodal means, which lay at 25 and 75% of the range, for both F1 and F2. This led to the mean F1 and F2 values listed in **Table 1**, which are quite close to those reported for /æ/ and /ε/ by Hawkins and Midgley (2005; see above in this section). The unimodal and bimodal distributions consisted of one and two Gaussian peaks, respectively, with standard deviations equal to 22 and 11% of the range, respectively. On the basis of these distributions, the F1 and F2 values for the 1800 training vowels were determined by a procedure described by Wanrooij and Boersma (2013), which approximates the intended probability densities of **Figure 1** optimally. The order of presentation of the 900 stimuli in the training was randomized separately for each infant. The inter-stimulus interval (the silent interval between the end of a stimulus token and the start of the next token) was 707 ms.

**Table 1:** F1 and F2 values (in ERB): means in the unimodal and bimodal training distributions, and values of the two test stimuli.

	Bimodal /ε/	Test stimulus 1	Unimodal	Test stimulus 2	Bimodal /æ/
F1	10.44	10.78	11.47	12.16	12.50
F2	20.37	20.14	19.68	19.22	18.99

#### In the test

In the test phase, infants were presented with two different stimuli, i.e., a standard and a deviant, repeated at most 2200 and 300 times respectively, depending on the infant (see section Procedure). Thus, deviants were presented at a rate of 12%. The F1 and F2 values of the test stimuli (**Table 1**) were determined by computing the intersections (circles in **Figure 1**) between the unimodal and bimodal distributions. In this way, the two groups of listeners came to the test phase with equal prior exposure (during training) to sounds in the region of the test stimuli, so that any difference between the groups observed in the test could not be attributed to differences in familiarity with the test stimuli. As during training the inter-stimulus interval in the test was 707 ms. In the test, minimally three standards (10 at the start of the test) appeared before each deviant. Apart from this constraint, the presentation of standards and deviants was randomized separately for each infant.

### PROCEDURE

Before training, the EEG cap with electrodes was placed on the infant’s head. During training and testing, infants were lying on the caregiver’s lap or in an infant seat beside the caregiver, in a sound-shielded room. Caregivers could watch a silent movie. Researchers in the adjacent room could hear caregiver and infant via loudspeakers, and observe them through a window. Researcher and caregiver did not know and could not consciously detect whether the distribution that was played during the training was unimodal or bimodal. The infant’s behavior was monitored and documented. Notes on behavior included the documentation of open or closed eyes, movement, fussiness, and pauses. Caregivers were asked not to interact with the infant, unless necessary to keep the infant quiet. In this case, recording was paused or (if it happened in the last minutes of the test) stopped. Excluding pauses, the training always lasted 12.1 minutes (900 training stimuli) and the test lasted between 29.7 and 33.6 minutes (between 2208 and 2500 test stimuli).

### CODING SLEEP STAGES

A factor that has to be considered when measuring MMRs is that during the relatively long experimental duration (viz., in the current experiment over 30 minutes, as compared to less than 10 minutes in behavioral distributional learning experiments) young infants tend to fall asleep (see also e.g., Friederici et al., 2002; He et al., 2009). It was therefore important to take a possible influence of sleep stages on MMR measurements into account. Infant sleep stages are usually divided into quiet sleep (QS), active sleep (AS), and wakefulness. Although some studies have not found any differences in neonates’ MMR amplitudes between different sleep stages (e.g., Martynova et al., 2003), there are two arguments to analyze data obtained in QS separately from data obtained during wakefulness for 2-to-3-month olds. First, for 2-month olds Friedrich et al. (2004) report a significantly larger positive MMR in QS than during wakefulness, as well as a preceding small negative MMR in wakefulness that was absent in QS. Second, sleep stages and the related EEG-patterns develop quickly into adult-like patterns already in the first 3 months of life (e.g., Crowell et al., 1982; Kahn et al., 1996; Graven and Browne, 2008), and the adult MMR during wakefulness differs from that during sleep, particularly during the successor of QS, non-rapid eye movement (NREM) sleep, where the response tends to disappear (e.g., Loewy et al., 1996; Loewy et al., 2000). In sum, there is at least some evidence that for 2-to-3-month olds the MMR in QS is different from that during wakefulness.

Sleep stages for each infant were determined on the basis of the infant’s behavior and the EEG. Stages in the EEG were coded in accordance with the AASM manual (Iber et al., 2007) and, because the manual’s age granularity is not precise enough to deduct recommendations for 2-to-3-month olds specifically, specifications for approximately the same age group from Crowell et al. (1982) and Niedermeyer (2005). Specifically, the stage was coded as “QS” when the infant’s eyes were closed and the EEG contained frequent spindles (i.e., more or less sinusoidal waves of 12 to 14 Hz, clearly distinguishable from background activity, and lasting at least 0.5 s; see also Rodenbeck et al., 2007) or apparent slow waves (with or without spindles) coming after parts with abundant spindling. The stage was coded as “AS” when the infant’s eyes were closed and the EEG featured transient muscle movements and low-amplitude mixed frequency activity. Finally, the stage was coded as “awake” when the eyes were open. When unequivocal identification was not possible (i.e., the eyes were closed but the EEG did not suggest QS or AS), the state was coded as “indeterminate sleep” (IS). A change of stage was not coded if the relevant changes in EEG and behavior lasted for less than 30 s (Iber et al., 2007).

It turned out that none of the infants stayed awake during recording. On average, they spent 13% of test time awake, 47% in QS, 1% in AS and 39% in IS. There were no significant differences in the time spent in each sleep stage between the four groups (four independent-samples Kruskal–Wallis tests, one for each sleep state, all *p*-values > 0.74).

For all subsequent analysis, we combined the three non-QS sleep stages (AS, IS, wakefulness) and labeled them together as “non-QS” (cf. Weber et al., 2004). As for AS, only three infants were in this stage for a short while (accounting for less than 2% of test time in any group), which is not surprising in the light of the rare AS onsets at 3 months of age and the relatively late expected start of AS after sleep onset as compared to the total test duration (Ellingson and Peters, 1980; Crowell et al., 1982); moreover, no reliable differences have been reported between MMRs during AS and MMRs during wakefulness in newborns (e.g., Cheour et al., 1998a; Kushnerenko, 2003). As for IS, we suspected that the infant was either well awake or drowsy, even though the eyes were closed, because the EEG in IS looked similar to that during wakefulness and did not contain any visual sign of QS. After combining the three non-QS variants, the sleep stages ended up being nearly equally divided between QS (47% of the time) and non-QS (53%).

### ERP RECORDING AND ANALYSIS

The EEG was recorded with a 32-channel Biosemi Active Two system (Biosemi Instrumentation BV, Amsterdam, The Netherlands) at a sampling rate of 8 kHz. Beside the 32 electrodes in the cap, two external electrodes were placed on the mastoids. After recording, the EEG was downsampled to 512 Hz (with Biosemi Decimator 86). Subsequent analysis was done in the computer program Praat (Boersma and Weenink, 2010). First, the EEG was tagged for sleep stages (see section Coding Sleep Stages). Then the EEG in each of the 32 channels was referenced to the mastoids (i.e., the average of the two mastoid channels was subtracted from each channel), “detrended” (i.e., a line was subtracted so that beginning and end of the channel signal were zero) and filtered (Hann-shaped frequency-domain, i.e., zero-phase, filter: pass-band 1–25 Hz, low width 0.5 – high width 12.5 Hz).

The subsequent analysis was done for QS and non-QS data separately, as follows. The EEG was segmented into epochs (32-channel ERP waveforms) of 760 ms duration (from 110 ms before to 650 ms after stimulus onset), for standard and deviant stimuli separately. For each epoch, a baseline correction was performed in each channel by subtracting from each (1-channel) ERP waveform the mean of the waveform in the 110 ms before stimulus onset. If after this an epoch (i.e., a 32-channel ERP waveform) still contained a peak below –150 μV or above +150 μV in one or more channels, the whole epoch was deemed invalid and rejected from further analysis. If after this fewer than 75 deviant epochs remained, the infant was rejected from the dataset for the relevant sleep stage. For each remaining infant, the standard and deviant responses were averaged separately, so as to obtain a mean standard ERP and a mean deviant ERP for each electrode. The infant’s 32-channel MMR waveform was obtained by subtracting the standard ERP from the deviant ERP.

### MMR ANALYSIS

In order to be able to submit the MMR measurements to statistical analysis, each infant’s MMR waveform was reduced to a small set of MMR amplitude values (see below in this section). To achieve this reduction, it was necessary to decide what electrodes and what time window(s) to include in the analysis. The literature that uses infant MMR analysis varies in these decisions and, relatedly, also in the reported results on where on the scalp the MMR was found and when the response occurred (see below in this section). In addition, the literature reports different polarities for the infant MMR (see below in this section). Thus, whereas the adult MMR is invariably a negative deflection (hence usually called a mismatch negativity, or MMN) that usually occurs between 150 and 250 ms after change onset, and is strongest at frontocentral electrodes (when the mastoids or the nose is used as a reference; for a review, see Näätänen et al., 2007), the infant MMR is much less defined in terms of *what* its polarity is, and *when* it occurs *where* on the scalp. We now explain our decisions on how these three aspects of the MMR waveform enter in our analysis.

As for the polarity of the infant MMR, it is sometimes reported as negative (e.g., Cheour-Luhtanen et al., 1995; Cheour et al., 1998b), sometimes as positive (e.g., Dehaene-Lambertz and Baillet, 1998; Dehaene-Lambertz, 2000; Carral et al., 2005), and sometimes as both negative and positive (e.g., Morr et al., 2002; Friederici et al., 2002; Friedrich et al., 2004). Regarding the variation in observed MMR polarities for infants across studies, we include both negative and positive values of individual infant’s MMR amplitudes in our analysis.

As for the location of interest on the scalp, some previous research selected only frontal electrodes (e.g., Morr et al., 2002) or frontal and central electrodes (e.g., Cheour et al., 1998b; Morr et al., 2002). When more posterior electrodes were included a significant infant MMR was sometimes reported only at frontal or frontocentral electrodes (Cheour-Luhtanen et al., 1995; Friederici et al., 2002; Friedrich et al., 2004), and sometimes also in more posterior areas (Cheour et al., 2002a; Van Leeuwen et al., 2008; He et al., 2009). As there is therefore some evidence that the infant MMR can be measured beyond frontocentral electrodes, our analysis includes not only six frontocentral electrodes (Fz, F3, F4, Cz, C3, C4), but also two temporal electrodes (T7 and T8); parietal and occipital electrodes were not included, because some infants had been lying on these electrodes. Following Cheour et al. (1998b), Morr et al. (2002) and Friedrich et al. (2004) we include the eight electrodes in the main analysis as a within-subject factor.

As for the chosen time window, the previous literature on infant MMR used various windows for vowels (e.g., 0–500 ms after stimulus onset in Cheour-Luhtanen et al., 1995; 200–500 ms in Cheour et al., 1998a) and various windows for 2- or 3-month olds (e.g., 0–1000 ms in Friederici et al., 2002; 200–600 ms in Friedrich et al., 2004; 100–450 ms and 550–900 ms in He et al., 2009). The only publication on vowels with infants in our age range (3-month olds: Cheour et al., 2002b) used a window from 150 to 400 ms. Regarding the reported variation, and because control of the Type I error rate dictates that analysis windows be chosen before the ERP results are seen, we had to choose in advance a window that includes at least the possible times at which the MMR can occur, namely a window running from 100 to 500 ms. In order to submit this window to an analysis of variance (ANOVA), we divide it into eight consecutive time bins of 50 ms each (Cheour-Luhtanen et al., 1995; He et al., 2009), and compute the average amplitude of the difference waveform in each bin as our measurement variable. To conclude, each infant’s MMR waveform is reduced to only 64 (8 time bins × 8 channels) MMR amplitude values.

### STATISTICAL ANALYSIS

To test whether there is a difference between unimodally and bimodally trained infants, while controlling for differences in the presented standard, we subjected the QS and non-QS datasets separately to an ANOVA with a mixed design (between-subject factors and repeated measures). The MMR amplitude was the dependent variable, Time Bin (100–150, 150–200, 200–250, 250–300, 300–350, 350–400, 400–450, and 450–500 ms) and Electrode (Fz, F3, F4, Cz, C3, C4, T7, and T8) were within-subject factors, and Distribution Type (unimodal vs. bimodal) and Standard Vowel ([æ] vs. [ε]) were between-subject factors. The design also included all possible interactions between the factors, up to the fourth order. To compensate for the double chance of finding results (separate QS and non-QS analyses) all tests employ a conservative *α* level of 0.025.

## RESULTS

**FIGURE 2:**
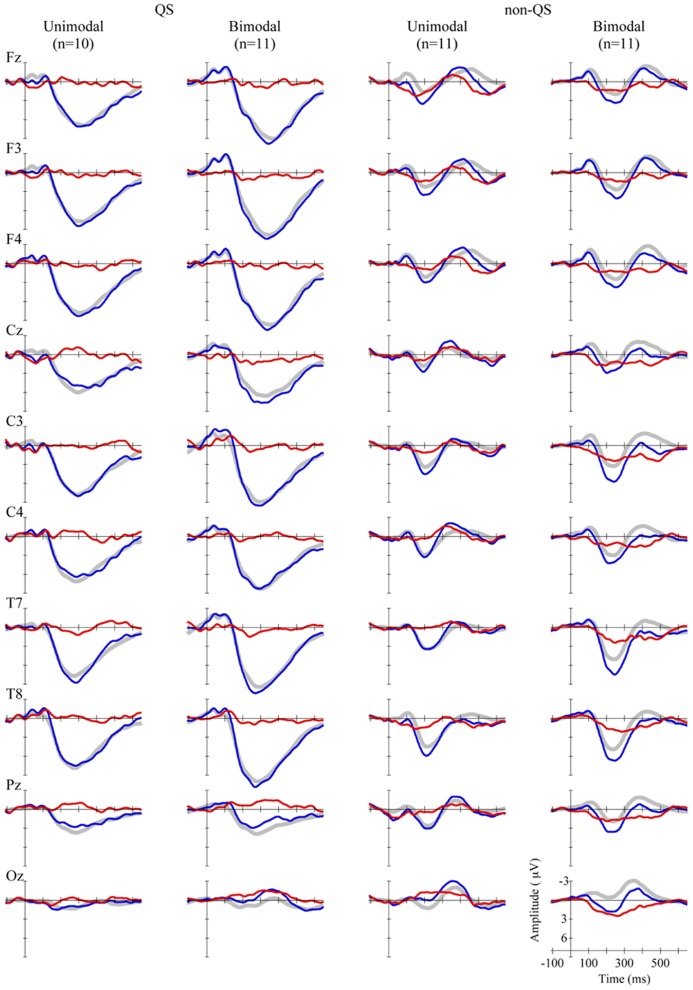
**Grand average standard (gray, thick curves), deviant (blue, thin curves), and MMR (red, thin curves) waveforms, at 10 electrodes (see rows), for unimodally and bimodally trained infants in QS (left two columns), and non-QS (right two columns)**.

The grand average waveforms for each Distribution Type (unimodal vs. bimodal) pooled over the two levels of the factor Standard Vowel are presented in **Figure 2**, for 10 electrodes. In line with previous research on 2-to-3-month olds, the standard and deviant ERPs contained prominent slow positive waves (e.g., Friederici et al., 2002; Morr et al., 2002; Carral et al., 2005; Shafer et al., 2011), and the ERPs in the QS data appeared large compared to those in the non-QS data (e.g., for 2-month olds: Friederici et al., 2002; for newborns: Pihko et al., 2004; Sambeth et al., 2009; but see Cheour et al., 2002a, for conflicting results).

For the QS data, the ANOVA on the MMR amplitude yielded significant results neither for the research question (main effect of Distribution Type: *p* = 0.88), nor for any other main effect (Standard Vowel: *p* = 0.23; Electrode: *F *< 1; Time Bin: *F *< 1), nor for any of the 11 interactions (all *p*-values > 0.07).

For the non-QS data, the ANOVA revealed a positive grand mean (+0.84 μV), with a 97.5% confidence interval (CI) that does not include zero (+0.35 ~ +1.33 μV), implying that on average Dutch 2-to-3-month old infants can discriminate the test vowels, and that vowel discrimination in these infants is reflected in a *positive* MMR. Regarding our specific research question, the analysis showed a main effect of Distribution Type (mean difference = +1.06 μV, CI = +0.08 ~ +2.04 μV, *F*[1,18] = 7.03, *p *= 0.016, $\eta_{p}^{2}$ = 0.28): across electrodes and time windows the bimodally trained infants had a higher positive MMR (+1.37 μV, CI = +0.68 ~ +2.06 μV) than the unimodally trained infants (+0.31 μV, CI = –0.38 ~ +1.00 μV), indicating that Dutch 2-to-3-month olds’ neural discrimination of [æ] and [ε] is better after bimodal than after unimodal training.

As for factors not directly pertaining to our research question, there was no effect of Standard Vowel (*p* = 0.98), so that we cannot state with confidence that one of the two combinations of standard and deviant vowel yields a higher MMR amplitude (and thus better neural discrimination) than the other combination. Further, the analysis showed no main effects of Time Bin (*F*[7*ε*,126*ε*, *ε* = 0.334] = 1.37, Greenhouse–Geisser corrected *p* = 0.27) or Electrode (*F *< 1). Thus, there was no support for a more positive or more negative MMR in any specific time window as compared to other ones within 100 and 500 ms, and at any specific electrode as compared to other ones among the frontocentral and temporal electrodes. Interestingly, we found a highly significant interaction effect between Distribution Type and Standard Vowel [*F*(1,18) = 20.22, *p* = 0.0003, $\eta_{p}^{2}$ = 0.53], which shows that the attested difference between unimodally and bimodally trained Dutch 2-to-3-month olds differs depending on the standard that they hear in the oddball test (see section Exploratory Results for the Four Groups).

### EXPLORATORY RESULTS FOR THE FOUR GROUPS

**FIGURE 3:**
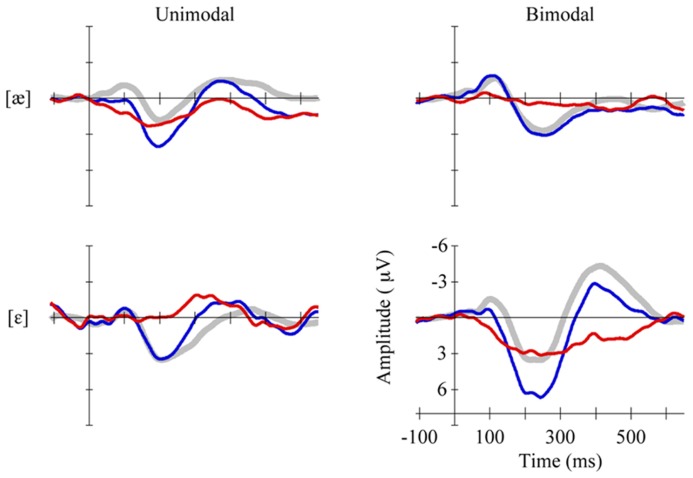
**Standards (gray, thick curves), deviants (blue, thin curves) and MMRs (red, thin curves) in non-QS, pooled across eight electrodes, per group (Unimodal [æ] top left vs. Bimodal [æ] top right, Unimodal [ε] bottom left vs. Bimodal [ε] bottom right)**.

To examine the responses of the four non-QS groups separately, we pooled the MMR amplitudes across electrodes and time bins in view of the lack of significant differences herein (see section Results). **Figure 3** shows the pooled MMR waveforms per group, and **Table 2** lists the corresponding averaged MMR amplitudes. The amplitude differed from zero significantly only for the Bimodal [ε] group (*p *= 0.004, uncorrected for multiple comparisons) implying that bimodally trained Dutch 2-to-3-month olds who are tested with standard [ε] and deviant [æ] can hear the difference between the two vowels.

**Table 2:** Mean MMR amplitudes (in μV) between 100 and 500 ms across eight electrodes per subgroup for non-QS data, with within-group standard deviations (SD; between parentheses), and 97.5% confidence intervals.

Distribution Type	Standard Vowel	*N*	Mean (SD)	Confidence interval	*t*	*p*
Unimodal	[ε]	6	–0.59 (0.86)	–1.71 to +0.52	–1.69	0.153
Unimodal	[æ]	5	+1.21 (1.23)	–0.71 to +3.14	+2.20	0.092
Bimodal	[ε]	5	+2.26 (0.83)	+0.97 to +3.55	+6.12	0.004
Bimodal	[æ]	6	+0.48 (0.80)	–0.55 to +1.50	+1.46	0.203

*Significance is tested against zero in four one-sample *t*-tests (without correction for multiple testing).*

**FIGURE 4:**
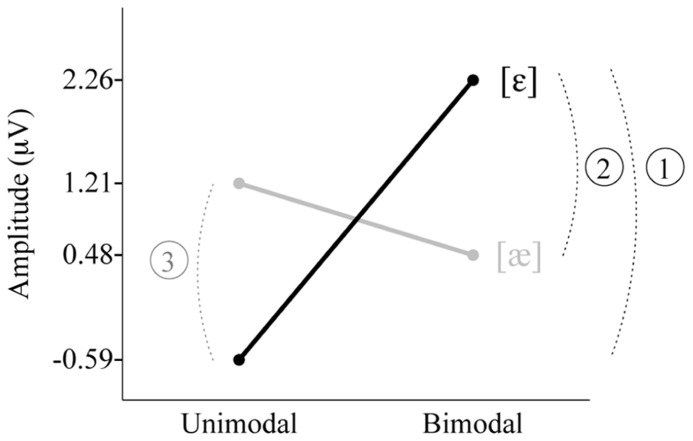
**Three *post hoc* significant differences in MMR amplitude between the four subgroups.** Unimodal [ε] (left black), Unimodal [æ] (left gray), Bimodal [ε] (right black), Bimodal [æ] (right gray). Note: Among the four amplitudes, only the one for the Bimodal [ε] group differed from zero significantly.

The individual group’s MMR amplitudes presented in **Table 2** are visualized in **Figure 4**. The interaction between Distribution Type and Standard Vowel, which was found in the main ANOVA for the non-QS data (see section Results), is clearly visible. We did the four relevant group comparisons, assuming equal variances for all groups (as in the ANOVA): Bimodal [ε] vs. Unimodal [ε], Bimodal [æ] vs. Unimodal [æ], Bimodal [ε] vs. Bimodal [æ] and Unimodal [æ] vs. Unimodal [ε] (technically, this was done via *post hoc* comparisons using Fisher’s Least Significant Difference in SPSS). The Bimodal [ε] group’s response was reliably more positive than that of the Unimodal [ε] group (see the arc numbered 1 and the black line in **Figure 4**; uncorrected *p *= 0.00008); this indicates that when the standard in the oddball paradigm is [ε] and the deviant is [æ], bimodally trained Dutch 2-to-3-month olds show better neural discrimination than unimodally trained infants. The difference between Bimodal [æ] and Unimodal [æ] was not significant (*p *= 0.21); thus, when the standard is [æ] and the deviant [ε], unimodally trained infants do not necessarily have higher response amplitudes. The Bimodal [ε] group’s response was greater than that of the Bimodal [æ] group (the arc numbered 2 in **Figure 4**; *p* = 0.005), suggesting that neural discrimination is easier for bimodally trained Dutch 2-to-3-month olds when the standard is [ε] and the deviant is [æ] than when standard and deviant are reversed. Conversely, the Unimodal [æ] group’s response was more positive than that of the Unimodal [ε] group’s response (the arc numbered 3 in **Figure 4**; *p* = 0.005), which suggests that neural discrimination is easier for unimodally trained Dutch 2-to-3-month olds when the standard is [æ] and the deviant is [ε] than when standard and deviant are reversed.

## DISCUSSION

The present study provides the first evidence for fast distributional learning in very young infants. The specific research question was whether Dutch 2-to-3-month old infants show larger mismatch responses, hence presumably better discrimination, of English [ε] and [æ] after bimodal than after unimodal training. This was answered in the affirmative with a *p*-value of 0.016. The age of 2 to 3 months is early enough for the distributional learning mechanism to be able to play a role in the transition from universal to language-specific speech perception, which has been observed to take place from 4 to 12 months.

This outcome extends previous research in two ways. First, fast distributional learning has now been attested at widely different ages, namely at 2 to 3 months (the present study), between 6 and 11 months (Maye et al., 2002, 2008; Yoshida et al., 2010; Capel et al., 2011), and in adults (Maye and Gerken, 2000, 2001; Gulian et al., 2007; Hayes-Harb, 2007; Escudero et al., 2011; Wanrooij et al., 2013; Wanrooij and Boersma, 2013). One can now hypothesize that the mechanism is available throughout life and can contribute to first and second language acquisition. Second, the ERP method has now been added to the set of methods by which distributional learning can be demonstrated. We needed the ERP method because of the young age of our participants, but this technique might have the general advantage over behavioral methods that it does not require the participant’s attention and that it taps the response process at a time when the response is still little influenced by the myriads of factors that contribute to the behavioral part of the response. An assessment of the general usefulness of the ERP technique, especially in comparison with behavioral techniques, has to await replication with more age groups, larger sample sizes and more phonological contrasts.

The ERP method potentially yields information on the scalp distribution and the timing of the responses. Our results, however, do not allow us to determine any precise scalp location or timing. This indeterminacy is not uncommon in studies on infant MMRs (see section MMR Analysis), and may be due to the more pronounced shapes of sulci and gyri in adults than in infants (Hill et al., 2010) and to the larger variability in MMR timing among infants than among adults (e.g., Kushnerenko, 2003). More location- or time-specific results can be expected at later ages.

This study detected an interaction between the type of distribution (bimodal vs. unimodal) in the training and the identity of the standard vowel ([ε] vs. [æ]) in the test (*p *< 0.001); *post hoc* exploration suggested that bimodally trained infants discriminated better if the standard was [ε] and unimodally trained infants discriminated better if the standard was [æ]. This confirms none of the three predictions that we derived from previous literature in the Introduction: the peripherality-related asymmetry predicted on the basis of Polka and Bohn (1996), namely that the MMR should be larger if the standard is [ε], was not found (main effect of Standard Vowel: *p* = 0.98); a prediction indirectly derived from the “natural referent vowel” hypothesis (Polka and Bohn, 2011), namely that the peripherality-related asymmetry should occur only in the unimodal group, was contradicted by our detection of the asymmetry in the bimodal group and the opposite asymmetry in the unimodal group; a prediction derived from the “featural underspecified lexicon” model (Lahiri and Reetz, 2010), namely that the MMR should be larger if the standard is [æ], was not confirmed (main effect of Standard Vowel: *p* = 0.98). None of the hypotheses in the literature predicted the asymmetry that we did find, and we cannot speculate on it before many more ERP results on asymmetries have been collected.

Given the effect of distributional training in the young infants tested, the question arises what the mechanism is: is there an enhanced discrimination in the bimodally trained infants (acquired distinctiveness), or is there a reduced discrimination in the unimodally trained infants (acquired similarity), or both? We cannot answer this question on the basis of our results, because time constraints prevented us from testing the infants’ perception before training. Also, a pre-test would have been an additional distributional training and could therefore have distorted the intended training distributions. Although to our knowledge MMRs for 2-to-3-month olds in response to similar small differences between vowels as between our test vowels (i.e., 1.38 ERB in F1 and 0.92 ERB in F2) have not been examined before, the acoustic difference between the test vowels was well above the discrimination threshold reported for 8-week old infants as measured behaviorally by high-amplitude sucking (Swoboda et al., 1976, 1978). On the other hand, the vowels in those studies were different, had different durations and were presented with different inter-stimulus intervals than in the current study, so that we cannot be certain that our 2-to-3-month olds discriminated the test vowels before training. Similarly, we cannot say if a potential perceptual ease of listening to the order [ε] – [æ] strengthened the effect of distributional learning for the bimodally trained infants and/or if a potential perceptual difficulty of listening to the opposite order weakened this effect.

One may wonder why the training–test paradigm works at all. After all, the test phase presents a (shrunk) bimodal distribution to the infants, and it can be expected that they continue to learn during the test, which lasts quite a bit longer (30 minutes) than what we call the “training” (12 minutes). The persistent influence of the training is possibly related to the much larger variability during training (900 different stimuli) than during the test (2 different stimuli). From other training paradigms it is known that a large variability in training stimuli can facilitate learning and could be instrumental in category formation (e.g., Lively et al., 1993). Future research is necessary to examine the persistence of short-term distributional learning over time.

With regard to the methodology of testing 2-to-3-month olds, the results highlight the importance of documenting sleep stages and analyzing QS data separately from non-QS data. In QS the MMR did not emerge, which is in line with the disappearance of the MMN in adult NREM sleep, and with the development of infant QS into an adult-like NREM in the first 3 months of life (see section Coding Sleep Stages), but in contrast to the lack of differences in the MMR between sleep stages in newborns (Martynova et al., 2003), and, for 2-month olds, to the *larger* MMR in QS than during wakefulness in Friedrich et al. (2004) and to the robust MMR in QS in Van Leeuwen et al. (2008). The many differences between these infant studies and the current study (if not simply due to chance) make it difficult to pinpoint the cause of this discrepancy. One difference from the studies mentioned is that the current study tested perception *after short-term training*. Thus, it may be that training effects were not yet sufficiently encoded in neural activation patterns to surface in QS. Alternatively, if infants who were in QS during the test, had already been in QS during the training, learning may have been hampered in QS as compared to non-QS.

We conclude that 2-to-3-month olds are sensitive to distributions of speech sounds in the environment. This is earlier than what has been shown in previous experiments with fast distributional learning, and earlier than the onset of language-specific speech perception. A linguistic interpretation of these results is that at 2 months of age infants already have a mechanism in place that can support the acquisition of phonological categories.

## Conflict of Interest Statement

The authors declare that the research was conducted in the absence of any commercial or financial relationships that could be construed as a potential conflict of interest.
